# Renewal of Life

**Published:** 1873-04

**Authors:** P. H. Hayes


### THE RENEWAL OF LIFE.

P. H. HAYES, M. D.

The first question to be asked about the promise or the performance of any man is, "how much *life* has he?" The mysterious question as to what life *is* in its essence, need not here be mooted. We speak of life as every one defines it, when they say such a man has got the "vim" in him, or such another man has got the "snap" in his composition.

The end of life is to enjoy it—wisely and well. But as to every object of happiness we enjoy therein, what and only what we bring the capacity to enjoy, and the ever underlying condition of capacity and enjoyment is the *health* of the faculties used.

The educated eye may be able to take in and enjoy marvellous beauties of landscape and water, of sky, cloud and sunshine; it may be ravished with the beauty of paintings and statuary, or with that which touches yet deeper depths in the soul, the beauty of the human face. But what is an eye good for whose nerve is already half dead, and can no longer respond except feebly to any impression of light? Where come in the beauties I have described, to the man who is shut up in a dark room with eyes inflamed, and who tells his surgeon that the very *thought* of *light* sends a gush of scalding tears down his cheeks. It will not quite do for the surgeon to say to this man, "surely, the light is sweet, and a pleasant thing it is to see the light of the sun."

The educated ear may be charmed to rapture with the sounds of music, nay, the songs of birds, the delicate sounds of the insect tribes, the voices of domestic animals may bring to the ear no small degree of pleasure, and lead us into a deeper and purer sympathy with nature. But what becomes of all this joy when disease shall make every sound a pain, a shock. What will you say of an ear to which the sweetest bird-song, or the music of a loving voice gives disturbance and agitation.

A man may have a mansion for his dwelling, wide, extended lands, with cattle upon a hundred hills, and be compelled to say, "all these profiteth me nothing while my *life* is worried out of me by the gout." What form of beauty in sight or sound can charm away the toothache, or what delectable dish can you find for a man with the colic?

There are then two kinds of *life;* healthy life and diseased life: or rather I should say, disease is not life at all, but partial death, a tint, a shadow of that change which pushed to its end destroys life.

Day by day we eat and say we live upon our bread and butter, our beef-steak and what not, but strictly speaking, we do not live upon food at all, but upon the blood which our digestive or blood-making organs make from our food. Moses said thousands of years ago that the "life of the flesh is in the blood," and physiology now brings out the full force of this idea, when we learn from it that every organ and tissue of the entire body feeds and lives at every instant upon the blood. Let the brain be deprived for one instant of blood, and the place turns dark, sounds are no longer heard, strength is gone, and the person falls to the floor in a fainting condition.

The digestive organs are the blood-making organs, and as is their life, or want of life. so is the quality and quantity of the blood they can make for the food of the organs and tissues. I have seen many persons who seemed to have as many diseases as they had organs in the body, and the real reason was that every organ was languishing for the want of good blood. Neuralgia is, in nearly all its forms, but an outcry of the nerves for more and better arterial blood. Rheumatism and gout, as the very words indicate, point to a humor or virus in the blood, and this humor or virus is really owing to a certain vice of digestion, whereby the humor is made in and with the blood. Scrofula and skin diseases, and many other chronic diseases, are owing to similar forms or types of depraved digestion or blood-making.

The blood of a strong man is about one-seventh part of his entire weight, so that if his total weight be one hundred and forty pounds, he will have twenty pounds of blood, and this blood flies through his body at such a rate that it goes the entire round of the circulation in about three minutes. We will suppose what cannot be far from the fact, that a man makes about one pound of blood in a day and uses up as the food of his body the same quantity. Waste and repair will then be equal and in twenty days there will not be an ounce of the blood left which he had twenty days before.

It is now easy to see how no chronic disease can be cured except as new and better blood is made, that from this a more pure nutrition of all the organs and parts of the body may take place, and *life* thus be created anew. This is a real renewal of life, for it must not be forgotten that the "*life* of the flesh," and it might be added of the spirit also, as to this tabernacle, is in the blood.

So then the digestive organs are a man's foundation, because they make the blood upon which the body lives. And so it is, that as is the digestion, so is the blood, and as is the blood, so is the body made from the blood, for the body is but the solid blood, and the the blood the fluid body. All things which minister to a sound and vigorous digestion, as out-of door exercise, proper bathing and rubbing, cheerful society, plenty of fresh air in living rooms, prosperous business, wisely selected food, a social way of eating, a mind at rest and at peace with itself, all these are of the greatest importance to a bright and sweet renewal of our life day by day.

A man should love his digestive organs. I had like to have said he should *pray* to them night and morning. I will say that he should sacrifice for them daily every thing which can disturb, distress, provoke, for in them may he see, if his eyes be not holden, the ark which contains the law of the covenant, which he can make with his own flesh for good or for evil.

But some one will say, if the blood can all be renewed in twenty days, why will not this time suffice for the cure of any disease whatever. This length of time is indeed often sufficient for the cure of acute diseases, a single renewal of the blood being quite sufficient for the renewal of the life, but in chronic diseases, where there is more or less old and permanent mal-digestion, only a little ground can be gained in this time, for each renewal of the blood only makes it a little better than it was before, yet does each renewal make the blood purer and richer than before, so that by and by a perfect blood is made and a healthy body is *pari passu*, built up from it. It must not be forgotten, moreover, that the digestive organs are themselves nourished in turn by the very blood they make for all other parts of the body, and of course with each renewal of the life of the blood, they are themselves fed and invigorated by a better blood, and thus and for this reason continually do their work better and better.

We are a nation of dyspeptics, and as a consequence our national disease is *exhaustion.*

How many enterprises fail from this cause, how many fortunes lost, how many aspirations for usefulness and fame cut off, how many drag out a gloomy life in this way it would be difficult to estimate.

> " 'Tis *life* whereof our nerves are scant,
> Oh! *life*, not death for which we pant,
> *More life*, and fuller, that we want."

